# Enhancing the Power of Genetic Association Studies through the Use of Silver Standard Cases Derived from Electronic Medical Records

**DOI:** 10.1371/journal.pone.0063481

**Published:** 2013-06-10

**Authors:** Andrew McDavid, Paul K. Crane, Katherine M. Newton, David R. Crosslin, Wayne McCormick, Noah Weston, Kelly Ehrlich, Eugene Hart, Robert Harrison, Walter A. Kukull, Carla Rottscheit, Peggy Peissig, Elisha Stefanski, Catherine A. McCarty, Rebecca Lynn Zuvich, Marylyn D. Ritchie, Jonathan L. Haines, Joshua C. Denny, Gerard D. Schellenberg, Mariza de Andrade, Iftikhar Kullo, Rongling Li, Daniel Mirel, Andrew Crenshaw, James D. Bowen, Ge Li, Debby Tsuang, Susan McCurry, Linda Teri, Eric B. Larson, Gail P. Jarvik, Chris S. Carlson

**Affiliations:** 1 Department of Public Health Sciences, Fred Hutchinson Cancer Research Center, Seattle, Washington, United States of America; 2 Department of Medicine, School of Medicine, University of Washington, Seattle, Washington, United States of America; 3 Group Health Research Institute, Seattle, Washington, United States of America; 4 Department of Biostatistics, University of Washington, Seattle, Washington, United States of America; 5 Department of Epidemiology, School of Public Health, University of Washington, Seattle, Washington, United States of America; 6 Biomedical Informatics Research Center, Marshfield Clinic Research Foundation, Marshfield, Wisconsin, United States of America; 7 Center for Human Genetics, Marshfield Clinic Research Foundation, Marshfield, Wisconsin, United States of America; 8 Essentia Institute of Rural Health, Duluth, Minnesota, United States of America; 9 Center for Human Genetics Research, Vanderbilt University School of Medicine, Nashville, Tennessee, United States of America; 10 Department of Biochemistry and Molecular Biology, Pennsylvania State University, University Park, Pennsylvania, United States of America; 11 Department of Biomedical Informatics, Vanderbilt University School of Medicine, Nashville, Tennessee, United States of America; 12 Department of Pathology and Laboratory Medicine, Perelman School of Medicine, University of Pennsylvania, Philadelphia, Pennsylvania, United States of America; 13 Department of Health Sciences Research, College of Medicine, Mayo Clinic, Rochester, United States of America; 14 Division of Cardiovascular Diseases, Mayo Clinic, Rochester, Minnesota, United States of America; 15 National Human Genome Research Institute, National Institutes of Health, Bethesda, Maryland, United States of America; 16 Program in Medical and Population Genetics, Broad Institute, Cambridge, Massachusetts, United States of America; 17 Department of Neurology, Swedish Medical Center, Seattle, Washington, United States of America; 18 Department of Psychiatry, School of Medicine, University of Washington, Seattle, Washington, United States of America; 19 VA Puget Sound Health Care System, Seattle, Washington, United States of America; 20 Department of Psychosocial and Community Health, School of Nursing, University of Washington, Seattle, Washington, United States of America; 21 Department of Medicine (Division of Medical Genetics), School of Medicine, University of Washington, Seattle, Washington, United States of America; 22 Department of Genome Sciences, School of Medicine, University of Washington, Seattle, Washington, United States of America; Queen's University Belfast, United Kingdom

## Abstract

The feasibility of using imperfectly phenotyped “silver standard” samples identified from electronic medical record diagnoses is considered in genetic association studies when these samples might be combined with an existing set of samples phenotyped with a gold standard technique. An analytic expression is derived for the power of a chi-square test of independence using either research-quality case/control samples alone, or augmented with silver standard data. The subset of the parameter space where inclusion of silver standard samples increases statistical power is identified. A case study of dementia subjects identified from electronic medical records from the Electronic Medical Records and Genomics (eMERGE) network, combined with subjects from two studies specifically targeting dementia, verifies these results.

## Introduction

Genome-wide association studies (GWAS) increasingly examine conditions for which cases are difficult or expensive to ascertain using traditional research approaches, such as rare adverse reactions to medications. On the other hand, as more health systems computerize their health data into Electronic Medical Records (EMRs), biobanks linked to EMRs would offer a rich source of potential cases, if suitable criteria for distinguishing cases were developed. Such “silver standard” EMR-derived criteria would likely have lower positive predictive value (PPV) of phenotype than the methods used in a traditional study of a disease, but researchers who used such a regime could augment the size of their studies for only the cost of data mining and informatics.

Immediately some practical concerns arise, such as whether inclusion of cases identified using a silver standard with a lower PPV might dilute the power of a study to detect a true genetic association. We address this concern by deriving an analytic expression for the power to detect an association using the chi-square test of independence, and confirm this expression by simulation. This analytic expression allows us to identify a subset of the parameter space *Ω* that characterizes a combined gold/silver study design that obtains increased power. The asymptotic expression and simulation framework are published in the R package *bimetallic,* available on cran.r-project.org, for researchers who wish to evaluate their own studies.

The increased power of this subset is then validated in real data from a GWAS of dementia risk from the Electronic Medical Records and Genomics (eMERGE) network [1]. In eMERGE, genome-wide Single Nucleotide Polymorphism (SNP) data were obtained from participants in five distinct healthcare systems, and linked to the longitudinal EMR data available at each site. At one site, participants with genome-wide SNP data were drawn from a prospective cohort study designed to detect incident dementia cases, with cognitive ability measured at two-year intervals after enrollment. The other sites had EMR data for their consenting participants. Because genotype data were available from the other sites, the only cost to using these data in a GWAS of dementia was the effort required for informatics and analyses. Thus, we have the opportunity of using EMR-derived cases from the other sites to supplement the research-grade cohort study.

The gold standard case and control data from the first site were used in the recently published multi-site Alzheimer's Disease Genetics Consortium GWAS of Alzheimer's disease [2]. In that study of gold standard cases and controls, nine different SNPs were associated with late-onset Alzheimer's disease (AD) at genome-wide significance levels (*P*<5×10^−8^), while one SNP had suggestive levels of association. Using these ten SNPs as positive controls, we compared the strength of association within eMERGE between analyses using solely the gold standard samples (n = 2526), or gold standard samples augmented with silver standard samples (n = 3369).

## Methods

### The power of a chi-square of independence with misclassification

Several authors have considered the effect of misclassification on estimation and inference in categorical responses. For chi-square tests on contingency tables, misclassification does not alter type-I error rates, but does reduce power [3]. The asymptotic power for chi-square tests with a given alternate hypothesis is known [4]. Others have applied this finding in the context of case-control genetic association studies under a constant rate of phenotype error and found an expression for the increase in sample size required to maintain constant power per percentage increase in misclassification [5].

### Chi-square tests

Let *G_ij_* be a 2 by 3 table of observed counts of genotypes given presence or absence of a dichotomous trait *i*. Let G_i_. be the marginal totals for the *i*th row (trait status) defined as
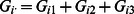
and similarly *G*._j_ is the marginal total for the *j*th column (genotype). Under the null hypothesis of independence between rows and columns, the expected number in cell *i*, *j* is given by 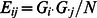 where *N* is the total number of counts in the table. Then the statistic defined by
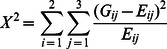
(1)is distributed chi-square with 2 degrees of freedom under the null hypothesis of no association.

### Distribution under alternative hypothesis

Under an alternative hypothesis of dependence on genotype frequency to trait status, *X^2^* is distributed non-central chi-square with a non-centrality parameter *λ* that depends on the difference between the case and control genotype counts. Edwards [5], adopting results originally described by Mitra [4], showed that if *M_1_* trait-present and *M_2_* trait-deficient individuals are sampled then
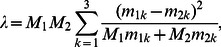
(2)where *m_1k_* and *m_2k_* are the (conditional) frequencies of genotype *k* in the trait-present and trait-deficient populations, respectively.

With a non-centrality parameter *λ* and a desired significance value of α, the power to detect an association is given by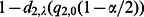, where 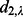 is the cumulative distribution function (CDF) of the non-central chi-square distribution with 2 degrees of freedom and 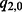 is the quantile function of the (central) chi-square distribution with 2 degrees of freedom. We note that power monotonically increases as *λ* increases.

Finding the power under a multipart phenotypic misclassification model is a matter of deriving the relationship of the genotypic disease risk and the misclassification parameters on *m_1k_* and *m_2k_*. To do that we need to specify parameters for phenotypic misclassification and genotypic disease risk models.

### A multipart phenotypic misclassification model

We draw a distinction between the terms “affected,” “unaffected,” ‘case,” and “control.” We consider affected/unaffected status to be a latent random variable *Z* that is unobserved in the silver standard subjects. Instead, a researcher observes *X*, the case or control criteria, for instance a set of criteria in an EMR. This leads to a 2×2 confusion matrix ***T*** for silver standard subjects, with elements giving P(Z|X) values in silver standard subjects. Denote the diagonal elements as φ and θ. These elements are equivalent to the positive and negative predicted values, respectively, of the EMR criteria.

A crucial assumption here is that *X* and genotype are conditionally independent given *Z*, i.e., genotype influences observed case/control status only through *Z*. Note that this implies that genotype errors are non-differential between cases and controls, a point which is further addressed in the Discussion. It also imposes a restriction on the path through which genotype affects the EMR phenotype. In particular, it could be the case that there are two conditions, *Z* and *Z*' which both are detected as *X*. In this case, the genotypes associated with *Z* or *Z*' will both appear to be associated with *X*, so one cannot conclude that an association between X and genotype is due to *Z* alone unless one can rule out the presence of the intermediary *Z*'.

When this conditional independence can be assumed to hold, then for gold standard subjects, the researcher directly observes Z, or equivalently, takes φ and θ to be unity. In practice this may not be a realistic assumption. Freeing φ and θ is an easy extension computationally, however, complicates exposition symbolically. A simple modification of the “setup.chisq” function in *bimetallic* allows an investigator to freely specify all classification rates, however we do not treat this possibility in this paper.

We express the numbers of gold and silver standard cases and controls primarily in terms of the number of gold standard controls, N_co_, and base the number of gold standard cases, silver standard controls and silver standard cases with the ratios *R*, γ_ca_ and γ_co_. Table 1 enumerates these relationships. With the numbers and ratios of cases and controls defined thusly, total numbers of trait-present subjects *M_1_* and trait-deficient subjects *M_2_* are given by 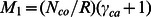 and 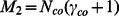.

**Table 1:** The parameter space Ω considered in simulation of power.

Parameter	Levels considered in simulations
R (number of gold controls per gold case)	1, 2, 4
γ_ca_ (# silver cases per gold case)	0, 1, 4
γ_co_ (# silver controls per gold control)	0, 1, 4
φ (positive predictive value of silver case)	0.6, 0.8, 1
θ (negative predictive value of silver control)	0.6, 0.8, 1
RR_AA_ (Relative risk in risk allele homozygote)	1†, 1.4, 3, 9
N_co_ (# gold controls)	200, 1000, 5000
m (risk allele frequency)	5%, 10%, 30%
k (disease prevalence)	0.1%, 1%, 30%
Genetic risk model	Dominant, recessive, or multiplicative

† denotes null model with no genetic risk.

The 2-by-3 matrix ***P*** gives the affected and unaffected conditional genotype frequencies. (In the section below, a genotypic disease risk model that could be used to populate ***P*** is described.) Then the permuted genotype frequencies in the silver standard population, the 2-by-3 matrix ***Q***, is given by the matrix product ***T***′ ·***P***, where ***T***′ is the matrix transpose of confusion matrix ***T***. This, for example, yields for the first cell in ***Q***:
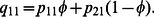
(3)


Then the case (*m_1k_)* and control (*m_2k_* ) conditional genotype frequencies in a mixed silver/gold study, may be expressed in terms of ***P***, ***Q*** and the phenotype misclassification model. The mixed frequencies *m_1k_* and *m_2k_* are merely weighted averages of ***P*** and ***Q*** given by
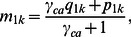
(4)


and
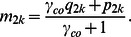



Combining equations 2, 3 and 4 and simplifying yields λ in terms of the multipart phenotypic misclassification model:
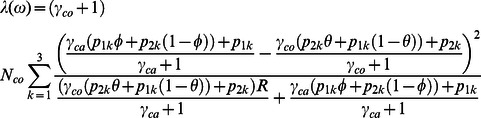
(5)


Using expression (5) and the fact that power is monotonic in λ allows the calculation of the marginal effect of adding a single silver standard case (or control), while holding other disease parameters fixed by finding, for example,



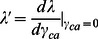
(6)If 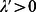, then power increases with the inclusion of silver standard cases.

### Genotypic Disease Risk Model

We adopt here a simplified version of Purcell's model for discrete traits [6], but any model for genotype frequencies conditioned on a dichotomous phenotype may be used. A bi-allelic locus in a diploid organism with genotypes AA, Aa and aa is assumed. For simplicity, it is assumed that Hardy-Weinberg holds at the margin for a locus with minor allele frequency *m*. Let the disease prevalence be given by P(Aff)  = *k* and the homozygous relative risk be given by 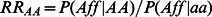.

We consider three models of allelic risk: dominant, recessive and multiplicative, corresponding to heterozygous relative risks equal to 1, RR_AA_ and RR_AA_^1/2^. Using the law of total probability, P(Aff|aa) may be expressed in terms of
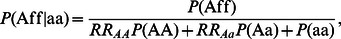
from which the rest of the genotypic conditional disease probabilities may be derived. Note that the model is over-specified, in that some parameter values induce P(Aff|AA) or P(Aff|Aa) >1, which we refer to as “unphysical” parameter values.

### Simulation Studies

We compared the power estimates derived in (5) to power in a multifactorial simulation of over 72900 different values in the 10-dimensional parameter space (4×3^9^ –5832 unphysical values). Of the 72900 combinations, 19683 correspond to models having no association between genotype and phenotype, allowing examination of the sampling distribution of X^2^ from expression 1 under a null hypothesis. Table 1 shows the parameter values considered in the simulation. The values in simulation were selected in an attempt to bound the set of plausible parameters values and thus exhaustively test the validity of the approximation, rather than being the most likely values an investigator would consider.

**Figure 1:**
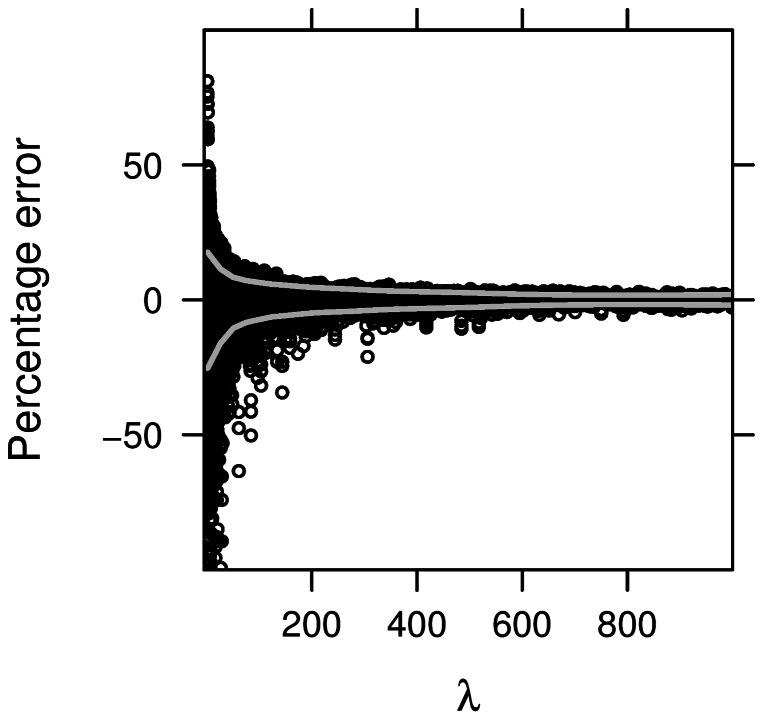
Error in asymptotic model. Percentage error in asymptotic model plotted against non-centrality, λ(ω), for various ω in Ω. Grey bands give approximate 90% bounds on percentage error, such that 90% of realizations in any λ-interval lie inside the region enclosed by the error bands.

We wish to evaluate the fidelity of the asymptotic approximation of X^2^ to its true sampling distribution, as determined through stochastic simulation. So we simulated 500 replicates of each ω in Ω and calculated X^2^. We calculated 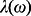, the value of the non-centrality parameter in equation 5 induced by ω. The 20^th^ percentile of all X^2^, 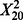 was adopted as the point of comparison. Since 80% of all realizations exceed this threshold, this percentile corresponds to the significance value achieved if power was fixed at 80% and type-I error allowed to vary. We compared 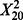 against 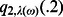, the 20^th^ percentile of the non-central chi-square distribution with two degrees of freedom and non-centrality parameter 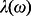. The percentage error of using the asymptotic approximation was calculated as
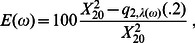
(7)and 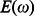 was plotted for various ω in Figure 1.

**Figure 2:**
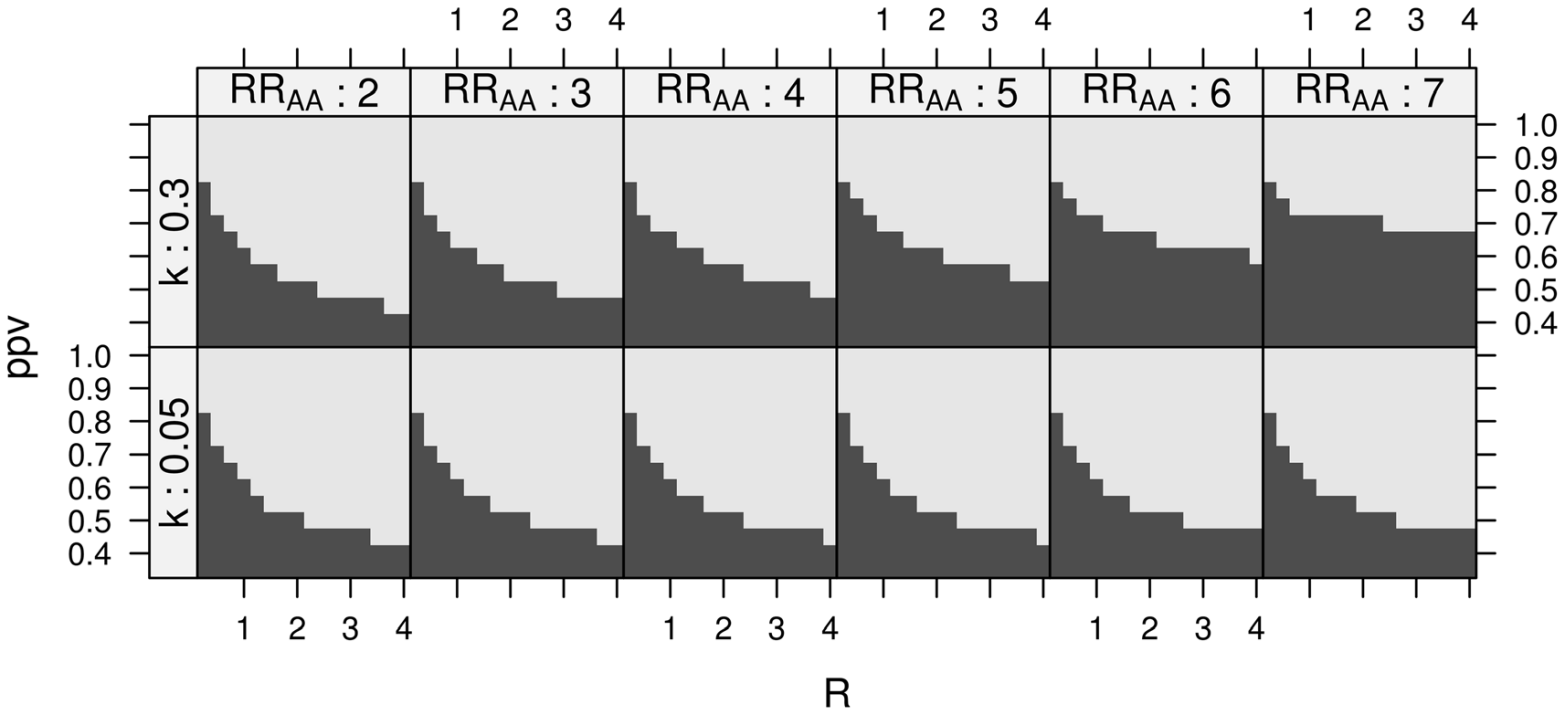
A subset with increasing power. Values of φ (y axis, between. 4 and 1) and *R* (x axis, values between 1 and 4) for which power is decreasing (dark) and increasing (light). Each panel shows a combination of prevalence, *k* by row (.05, .3) and homozygous relative risk RR_AA_ by column, range 2–7. Prevalences <.05 are not shown here because of similarity to the panels for *k*  = .05.

In figure 2, the sign of λ', *ie*, the change in power, for a representative subset of the parameter space is depicted.

### Power at ADGC-identified SNPs using silver standard cases

In order to empirically validate our models, we used the genotypes of gold and silver standard eMERGE participants (described below) to compare power under expression 5 to a bootstrapped estimate of power under the 2×3 chi-square test of independence. Ten loci identified in two recent GWAS of AD [2], [7] were considered. Two loci had evidence of association using a chi-square test for independence between genotype and phenotype at P<.05 using gold standard participants (N = 2526) alone.

We then considered a series of hypothetical studies including both gold and silver standard participants in varying ratios. Genotypes from the combined studies were resampled many times, to approximate the sampling distribution of X^2^ statistics through bootstrapping.

More exactly, to compute the bootstrapped estimate, we sampled with replacement the genotypes at the locus in question, conditional on the genotypes belonging to the set of gold standard dementia cases, silver standard cases or gold standard controls in our study. Various ratios of gold and silver standard cases were used, corresponding to different *γ_ca_* and *R* in the model described above. 1000 replicates per locus per ratio-combination were found to calculate 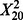, the 20^th^ percentile all X^2^ statistics.

Then we compared 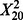 to the asymptotic value, 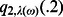, by making assumptions about disease prevalence (*k*  = 0.13), risk model (multiplicative) and the PPV of the EMR criteria (φ = 0.7). The minor allele frequencies and odds ratios (ORs) assumed are taken from the replication cohort from and are given by table 2, except for rs2075650, for which a homozygous OR of 3.2 was assumed.

**Table 2:** Parameters to estimate asymptotic power and results from association study for eMERGE gold standard (N = 2526) cohort.

SNP	Nearest Gene	Het OR	MAF	Gold P
rs4938933#	MS4A4A	*0.88*	0.39	0.18
rs9349407#	CD2AP	*1.12*	0.27	0.16
rs11767557	EPHA1	*0.87*	0.19	0.18
rs3865444	CD33	*0.89*	0.3	0.23
rs6701713	CR1	*1.16*	0.2	0.02
rs1532278#	CLU	0.89	0.36	0.59
rs7561528	BIN1	*1.17*	0.35	0.48
rs561655#	PICALM	*0.87*	0.34	0.58
rs2075650	APOE	2.2	0.12	<1e-21
rs3752246#	ABCA7	1.13	0.19	0.40

Abbreviations: MAF, Minor Allele Frequency; Het OR, Heterozygous Odds Ratio; Gold P, P value in gold standard participants.

#denotes imputed loci.

**Figure 3:**
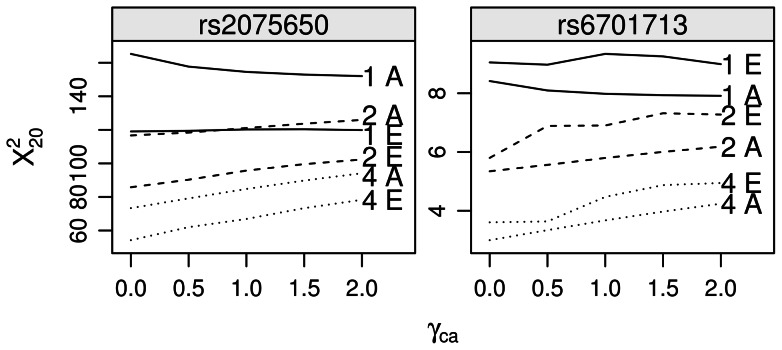
Empirical power and asymptotic power. Comparison of empirical power (E) to asymptotic (A) for various *γ_ca_* and *R* = 1 (solid line), *R* = 2 (dashed), *R* = 4 (dotted) at two loci that nominally replicate in the gold standard subset. Power is shown as 20^th^ percentile of X^2^ statistics over 1000 bootstrapped replicates for empirical graphs or as 20^th^ percentile of the chi squared distribution for asymptotic graphs, with non-centrality determined from genotypic disease model given in table 2.

Results are presented in figure 3 below.

### Gold standard cases and controls

Participants with gold standard case and control status were drawn from a study and its planned successor based at Group Health Cooperative in Seattle, a large health maintenance organization. The initial study provided only cases. The University of Washington/Group Health Cooperative Alzheimer's Disease Patient Registry (ADPR) provided 243 cases of AD. Case identification methods of the UW/GHC ADPR have been published [8]. Potential early AD cases were identified from a number of clinical data sources and were brought in for thorough neuropsychological and neurological examinations, from 1987 to 1996. Dementia was diagnosed using Diagnostic and Statistical Manual (DSM) III-R or IV criteria [9], and AD using NINCDS-ADRDA criteria [10]. DNA was extracted as previously described [11].

The succeeding study, the Adult Changes in Thought (ACT) study, provided both gold standard cases and all of the gold standard controls used in this study. Both studies have the same grant number and PI (U01 AG 06781, Eric Larson, PI). ACT includes urban and suburban elderly populations from a stable health management organization [12], [13]. ACT began as a cohort of 2,581 cognitively intact participants older than 64 years. Later, an expansion cohort (n = 811) was enrolled. Currently the study employs a continuous enrollment strategy to maintain approximately 2000 at-risk persons in the study, resulting in a total enrollment of 4,600 participants as of June 2012. ACT has an exemplary Completeness of Follow-up Index (95.6%) [14].

ACT participants are administered the Cognitive Abilities Screening Instrument [15] at baseline and again every 2 years. A 2-stage screening process is used to identify dementia cases; Cognitive Ability scores ≤85 prompt a dementia evaluation. Informant, subject, or staff reports of cognitive difficulties also trigger evaluation. The 2^nd^ stage diagnostic examination includes neuropsychological testing and a neurological exam. Medical records are abstracted for standard labs and neuroimaging reports. If any are unavailable in the prior year they are requested. These data are used to complete DSM-IV diagnostic criteria for dementia and subtypes [9], NINCDS-ADRDA criteria for AD [10], and criteria for vascular dementia [9], [16], [17], [18]. All clinical data are reviewed at a consensus conference. These procedures are unchanged –and are conducted by the same personnel – as the ADPR case finding methods described earlier. ACT dementia and AD incidence rates are consistent with those found worldwide [12].

The IRB granted a waiver of consent for eMERGE for deceased ACT and ADPR participants, but required re-consent for living participants. We asked participants for consent by mail; participants with an imminent visit we asked in person. Participants were very receptive; we had an acceptance rate of 86%. We also made a great effort to obtain consent from the legally authorized representative for participants who had developed dementia.

There were 391 individuals from ACT included in eMERGE genotyping with probable or possible AD, 121 with other forms of dementia, and 2,065 controls without dementia.

### Ethics

The institutional review board of the Group Health Research Institute approved this study. Participants from the gold standard cohort gave written consent for genetic analyses either under the auspices of the Genetic Differences study (R01AG007584, Walter Kukull, PI) or gave written consent as indicated above. Participants in the silver-standard cohorts (described below) gave consent as follows: Marshfield [1] and Mayo [19] participants gave written consent upon enrollment in their respective studies or biobanks. Vanderbilt's participants gave written consent to entry in the BioVU biobank on their consent-to-treatment forms before blood is drawn for clinical purposes, or could opt out at that time [20].

### Derivation of an EMR-based silver standard Alzheimer's case definition

We used data from the ACT study to develop an EMR-derived silver standard case definition [21]. The development set consisted of 537 cases meeting DSM-IV criteria for dementia and 2915 dementia-free controls. We divided the development set into training and test sets to avoid overfitting. No participants in our development set were under 65 years old, but we also set an *a priori* exclusion on participants younger than 65 years at first ICD-9 code/medication fill to screen out early onset AD participants that might be found in younger populations.

We considered several sources of data for the model, including ICD-9 codes for Alzheimer's disease and dementia, specialty of the healthcare provider using those codes, other events at visits producing those codes such as neuroimaging tests or laboratory tests used to diagnose dementia subtypes (e.g. TSH or B_12_ levels), and pharmacy data for medications used to treat dementia such as memantine, donepezil, galantamine, or rivastigmine.

We considered data for each case up to the date they were evaluated for dementia by the ACT study. We did this because the ACT study notifies the primary care providers of enrollees whom the study identifies as having dementia, and this notification likely influences subsequent medical care and resulting ICD-9 codes. This truncation mechanism limits the severity and overtness of the dementia cases. As dementia progresses, it becomes more clinically obvious, and less likely to be missed. Our choice to truncate clinical data at the time of dementia diagnosis suggests that our PPV will be a conservative estimate of the accuracy of silver standard case criteria in other settings, where cases could have any level of dementia severity.

Our first priority for the EMR-derived case definition was to maximize the PPV of our case definition, and the second priority was to maximize sensitivity of the definition. The criteria “five or more qualifying ICD-9 codes, or one or more Alzheimer's medication fills” provided the highest PPV and sensitivity in our training set. In the test set, these criteria yielded a PPV of 0.73 and a sensitivity of 55%. The specific drugs and ICD-9 codes are indicated in Document S1.

For the purposes of power calculations, we assume that the PPV found in the ACT study cases serves as a lower bound on the PPV in the other sites when they applied the algorithm for the reasons detailed above. Note that there may be differences between silver standard sites in the PPV. This differential classification will not impact our power calculations as long as the PPV used in the calculations is indeed a lower bound for all of the silver standard sites.

### Source of silver standard cases

Three other sites within eMERGE were selected to implement the EMR-case definition on the basis of the participant demographics at the sites. The Marshfield Clinic Personalized Medicine Research Project, the Mayo Clinic's biobank and Vanderbilt University's biobank BioVU have been described previously [1],[19],[20],[22]. Data from Marshfield included a subset (N = 153) that had previously been evaluated, also with an EMR-based algorithm. As these individuals were identified from a clinical delivery system rather than a research study design that characterized our gold-standard cases, we treated the Marshfield cases, including the subset previously re-evaluated as published in [23], as silver-standard cases in the analyses presented here. The number of cases contributed from each of these sites is listed in table 3.

**Table 3:** Participants by institution and genotyping center in combined gold/silver standard association study.

Batch	Genotyping center	MF	VU	GH	MAYO	Total
1	CIDR	222	270	2791	0	3283
2	CIDR	231	0	0	0	231
3	BROAD	0	0	0	265	265
Total		453	270	2791	265	3779

Abbreviations: MF, Marshfield Clinic Personalized Medicine Research Project; VU, Vanderbilt University BioVU; GH, Group Health/University of Washington Adult Changes in Thought and Alzheimer's Disease Patient Registry; MAYO, Mayo Clinic biobank; CIDR, Center for Inherited Disease Research.

Since the negative predictive value of EMR proxies for absence of dementia appeared to be poor, we did not pursue adding silver standard controls. The high prevalence of dementia in elderly populations means that the specificity of any proxy must be quite high for the negative predictive value to be acceptable. In conditions with lower background prevalence and more dramatic clinical features likely to be picked up in the course of routine clinical care, searching for silver standard controls would be more reasonable.

### Genome-wide SNP methods

The Group Health, Mayo Clinic, Marshfield, and Vanderbilt samples were genotyped at the Center for Inherited Disease Research at John Hopkins University or the Broad Institute of Harvard and the Massachusetts Institute of Technology; 84% of samples were genotyped in batch 1, which we treated as the primary dataset for the purposes of quality control. The samples in batch 1 were block randomized by phenotype and study center, with assay plate as the blocking factor. All sets and samples were genotyped on the Illumina Human660W-Quadv1_A array. Genotypes were called by the respective genotyping centers using the software package GenomeStudio.

We undertook an extensive quality control process, using software packages PLINK v1.07 [24] and R [25] and following published protocols [26]. We began with a total of 3,779 unique samples. We tested for and removed sex-discrepant samples, samples with significant kinship and samples with non-European ancestry [27]. Population structure appeared to be well controlled (genomic control coefficient 1.003). All samples exceeded a call rate of 98%. After the above filtering steps, a set of 672 gold standard cases, 1854 gold standard controls and 843 silver standard cases remained (total n  = 3,369, 89% of the original samples).

We also examined the quality of individual SNPs with several metrics. We received genotypes at a total of approximately 560,000 SNPs from the genotyping centers. We removed monomorphic SNPs, SNPs with call rates lower than 98%, and SNPs with more than 1 replicate discrepancy. We screened for technical artifacts in the genotype clustering between the primary and secondary datasets by using common controls.

For some loci in our bootstrap power comparison (noted in table 2), we imputed the value of the locus because it was not genotyped directly. We used the software package IMPUTE2, with 120 European samples from the 1000 Genome Project [28] and a multi-ethnic set of 1920 samples from HapMap phase 3 as reference panels [29], [30].

The studies are available from dbGAP under accession number phs000234.v1.p1.

## Results

### Simulation study

Figure 1 shows E(ω), the percentage error of 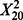  between the sampling distribution imposed by (5) and the simulated, true distribution of X^2^ statistics under a wide array of values considered in Ω. For small values of *λ*, the asymptotic distribution does depart from the true distribution. However, as λ increases, the error decreases and the asymptotic distribution better approximates the true distribution. Thus for larger sample sizes, one may use the analytic expression for slope of the asymptotic power function, as available with the function “dlambda” in *bimetallic* to test the benefit of including a silver-standard case under a desired study design.

In the null models, a two-sided Kolmogorov-Smirnov test finds a decisive lack of fit to 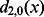. (P<*10−^16^*) over all the *7.8*×*10^6^* realizations of *X^2^*. This is indicative of the asymptotic convergence of *X^2^* to 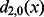. Indeed, as the effective sample size of the simulation 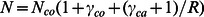 increases, the goodness of fit increases, such that there is no evidence of departure from 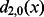 (Kolmogorov-Smirnov P = 0.8) for N ≥4000 evenly split between cases and controls (194,400 realizations of *X^2^* considered). Figure S1 suggests that under simulation type-I error is maintained at nominal levels: the P-values from null models are uniformly distributed.

Figure S2 illustrates bias in point estimates of allelic ORs under the misclassification model. In particular, ORs are biased towards one. This bias is a function of φ and γ_ca_. However, since estimates of ORs in GWAS are biased (away from one) inherently when the samples used to ascertain significance are also used to estimate ORs [31], we do not believe this is a practical impediment for investigators who wish to use the combined study designs we describe here for discovery of linked loci. We do recommend that investigators locate an independent replication set (measured without error), or utilize double sampling methods previously described for the purposes of calculating ORs [32], [33].

### A subset of Ω for which power is increasing in γ_ca_

If (6) is positive, power increases with the addition of silver standard cases to the analysis. We demonstrate a range of disease/diagnosis models for which this is true in Figure 2. We examine here a multiplicative risk model (RR_AA_  =  RR_Aa_^2^) and a risk allele with population frequency *m = 0.3,* and plot positive predictive value of silver standard diagnosis (φ) versus gold control:gold case ratio (*R*) for combinations of disease prevalence (k) and relative risk in risk homozygotes (RR_AA_), but note that these results hold qualitatively for many other risk models and *m* (data not shown).

The most important relationship observed is between *R* and φ. The inclusion of silver standard cases with relatively low PPV (φ) can still increase the power of a study if the ratio of controls to cases (R) is relatively high. Other minor features of the model are that smaller values of RR_AA_ allow smaller values of φ at large *R*, and that risk models that result in high penetrance SNPs (such as the *k*  = 0.3 and RR_AA_ >5 panels) require larger φ for all *R*.

### Application to previously identified SNPs using silver standard cases

Figure 3 plots the 20^th^ percentile of X^2^ statistics for two AD risk loci under various ratios of gold and silver standard cases and controls, described above in Methods. These loci replicate at P<.05 in the gold standard case/control set. The “empirical power” curves (suffixed with E) are determined by bootstrap at each abscissa of *γ_ca_*. The asymptotic power (suffixed with A) is determined by 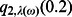, with ω parameters given by table 2. Power is plotted for ratios of *γ_ca_* (abscissa) and for control:case ratios *R* of 1, 2 and 4.

Although power is systematically overestimated at rs2075650 and underestimated at rs6701713, the shape of empirical curves matches the shape of asymptotic curves: higher *R* yield marginally greater returns to *γ_ca_*, and for *R* = 1, power is reduced by including silver standard cases. Some reasons for systematic deviation of empirical power from asymptotic power are described in the Discussion below.

## Discussion

Samples with high-density genotyping data available across multiple phenotypes in an EMR are a potentially valuable resource for genomic association studies. Our study demonstrates that even for a disease with a relatively low PPV of EMR diagnosis, there are realistic scenarios for which the addition of silver standard participants boosts the power to detect a true association. We find that there is no inflation of type-I error under such scenarios. There is very good agreement between asymptotic and simulated power, and good agreement between bootstrapped and asymptotic power. However, estimated asymptotic power deviated modestly from the true power to detect a disease. The genetic risk model is not identifiable from the data alone, so these deviations may stem from incorrect assumptions on the mode of inheritance (dominant, recessive, or otherwise). An overestimate of power could be indicative of phenotype or genotype error in our GWAS samples. Although there is much discussion of bias in ORs derived from GWAS and factors that inflate the type I error rate [31], [34], we know of no study comparing observed to expected power for well-characterized risk loci.

We find that two parameters should have greatest influence on investigators contemplating augmentation of their GWAS with silver standard samples. Of greatest import is the control:case ratio for gold standard phenotypes, *R*. When excess gold standard controls are available (high *R* ratio) the inclusion of silver standard cases yields the greatest improvements in power. Inversely, at small *R*, scenarios exist such that incorporating additional silver standard cases reduces power. Of secondary importance is the PPV of the criteria used to identify silver standard cases. We show in figure 2 that there exists a minimum PPV for silver standard cases to result in positive power for hypothesized small effect sizes. This minimum is subject to other parameters of the risk model, but is typically around 0.6.

The evidence presented here that differential error in phenotype classification has limited and predictable effect on hypothesis testing must be tempered by the fact that silver-standard predictive values are unlikely to be known without access to a cohort measured along both dimensions. Indeed, as described in the Methods, the existence of the GHC cohort, measurable by both criteria was intrinsic to the development of the EMR criteria. However, since it is the PPV (as opposed to the sensitivity or specificity) that needs to be estimated, conducting a secondary chart review or additional diagnostic tests in a subset of the silver standard population will suffice. There are successful examples of this approach described for peripheral arterial disease [19], diabetes [35] and other phenotypes [36].

Although this manuscript suggests that combining cases from multiple studies can yield improvements in power, we recommend unified genotyping of the experiment, so that phenotype (eg, gold and silver standard cases and controls) may be randomized across nuisance factors (like plate or chip version). Caution must be exercised when such randomization cannot be performed, since differential genotyping error between phenotypes can result in not only reduced power, but also spurious findings [37], [38].

In practice, additional data will need to be collected unless the experimental design is flawless. This data could take the form of double sampling all genotypes in a subset of subjects, which allows the estimation of error rate for each locus and application of tests that efficiently use such double sampling to correct for differential genotype error [39] This additional data could also simply be the validation of interesting findings via an alternate, lower throughput technology in which appropriate experimental design is applied.

We readily acknowledge that chi-square tests are unlikely to be optimal in many GWAS. However, we believe a characterization of their power is useful due to the existence of closed-form formulae. This makes it feasible to consider a variety of scenarios, and to examine power at the margin of an additional sample, with the expectation that the qualitative results will continue to hold in more complex models. Replacing the chi-squared test with logistic regression in a representative subset of the parameter space considered in the simulation study supports this assertion. In 88% of scenarios, the predicted slope of the power curve given by expression 6 matched the observed change in power after adding silver standard cases. See table 4.

**Table 4:** Concordance between power in logistic regression and slope of power curve given by equation 6.

R	φ	RR_AA_ = 1.4	RR_AA_ = 3	RR_AA_ = 9
1	0.6	1	1	1
1	0.8	0.75	1	1
2	0.6	0.33	0.83	1
2	0.8	0.92	1	1
4	0.6	0.75	0.92	0.83
4	0.8	0.92	1	1

Proportion of scenarios in which observed change in power agreed with predicted slope of chi-square power. The observed change in power is calculated as the sign of the difference of the median likelihood ratio statistic at γca  = 0 and at γca  = 1. 160 parameter values were considered, a subset of the parameter space described in table 1. 500 realizations at γca  = 0 and at γca  = 1 of each combination were undertaken to find the median likelihood ratio statistics.

In conclusion, the re-use of samples with available high-density genotype data and rich phenotypic data (such as in an EMR) can cost-effectively enhance statistical power under a range of realistic scenarios.

## Supporting Information

Figure S1 **P-values from null models in simulation are approximately uniformly distributed.**
(EPS)

Figure S2 **Bias in point estimates of odds ratio for various φ and γca with k = .01, R = 4, m = .3, RR_AA_  = 3 and a multiplicative disease risk model.**
(EPS)

Document S1 **ICD-9 Codes and Medications defining silver standard cases from EMR.**
(XLS)
